# Associations of Acetic Acid Intake with Protein and Vitamin Intake Estimated via a Food Recording Application

**DOI:** 10.3390/nu16172977

**Published:** 2024-09-03

**Authors:** Kanako Deguchi, Joto Yoshimoto, Risako Yamamoto-Wada, Chihiro Ushiroda, Kotone Yanagi, Mikiya Kishi, Hiroyuki Naruse, Katsumi Iizuka

**Affiliations:** 1 Department of Clinical Nutrition, Fujita Health University, Toyoake 470-1192, Japan; kanasakuran@gmail.com (K.D.); risako.wada@fujita-hu.ac.jp (R.Y.-W.); chihiro.ushiroda@fujita-hu.ac.jp (C.U.); 2 Central Research Institute, Mizkan Holdings Co., Ltd., 2-6 Nakamura-Cho, Handa-Shi 475-8585, Japan; yoshimoto_jyoutou@mizkan.co.jp (J.Y.); mkishi1@mizkan.co.jp (M.K.); 3 Health Management Center, Fujita Health University, Toyoake 470-1192, Japan; yanagi-k@fujita-hu.ac.jp; 4 Department of Medical Laboratory Science, Fujita Health University Graduate School of Health Sciences, Toyoake 470-1192, Japan; hnaruse@fujita-hu.ac.jp

**Keywords:** food recording application, acetate intake, vitamin B1, niacin, vitamin B12

## Abstract

A conventional questionnaire-based assessment of acetic acid intake is based on the intake of seasonings such as mayonnaise, which could thereby lead to an underestimation. We here determine the relationships of acetic acid intake with nutrient intake estimated using a food recording app (Asken) based on meal recipes. A total of 141 individuals (48 men and 93 women) used the app for at least 7 days per month. The mean daily intake of acetic acid was 0.16 ± 0.19 g and the mean frequency of acetic acid intake was 2.77 ± 1.66 days per week. A multivariate regression analysis adjusted for age, sex, BMI, and energy intake revealed that the amount of acetic acid consumed was significantly and positively associated with the intake of protein (11.9 (5.1, 18.6), *p* < 0.001), cholesterol (80.7 (4.5, 156.9), *p* = 0.04), and all vitamins except vitamin K. The frequency of acetic acid intake was significantly and positively associated with protein (1.04 (0.20, 1.87), *p* = 0.015), vitamin B1 (0.3 (0.02,0.5), *p* = 0.031), niacin (0.5 (0.04,1.0), *p* = 0.032), and vitamin B12 (0.4 (0.1,0.7), *p* = 0.002) intake, suggesting that individuals who frequently consume acetic acid tend to consume more protein and some vitamins. Thus, the amount and frequency of acetic acid may reflect protein and vitamin intake.

## 1. Introduction

Acetic acid (strictly, ethanoic acid) is the unionized form, CH_3_CO_2_H, while acetate (strictly, ethanoate) refers to the ionized form, CH_3_CO_2_^−^. Acetic acid is a main component of vinegar, mayonnaise, and ketchup and is an ingredient in seasonings [1,2,3]. Commonly consumed vinegars contain between 4% and 8% acetic acid [1,2,3]. Sodium acetate is also used as a food additive because it maintains pH, improves taste, and extends the shelf-life of food; however, the amount of sodium acetate added in foods is much smaller than the amount of acetic acid that is normally added [1,2,3]. Acetic acid is found mainly in rice balls, steamed fish paste, bread, processed food (croquettes, hamburger patties, meatballs, dumplings, etc.), dairy products, dried pastas, salt substitutes, coffee, and coffee substitutes. Thus, acetic acid and acetate are found in various foods as a seasoning and food additive.

Early records from China, the Middle East, and Greece describe vinegar for medicinal purposes: a digestive aid, an antibacterial balm to dress wounds, and a treatment for cough [2,3]. Acetic acid is also considered a healthful substance, but there are few reports on acetic acid intake. Several studies have examined the relationship between the intake of vinegar containing acetic acid and obesity status. Some have reported that 15 mL of vinegar (0.75 g acetate) inhibits weight gain in humans by suppressing appetite [4]. Four of the six short-term studies reported that vinegar consumption suppressed appetite, whereas none of the long-term studies were able to reproduce these results [5]. Some have reported that daily vinegar intake in amounts of ~10–30 mL (~2–6 tablespoons) appears to improve the glycemic response to carbohydrate-rich meals; however, few studies have investigated the chronic effects of vinegar intake [6]. Some clinical trials using orally ingested acetic acid have also shown various effects, such as lowering blood pressure [7], suppressing increases in fasting [8] and postprandial [9] blood glucose, and reducing feelings of depression [10]. Therefore, estimating the amount of acetic acid consumed in the diet may lead to new discoveries about the functions of acetic acid. In some animal studies, acetic acid has also been shown to protect against obesity and glucose intolerance by increasing lipolysis in adipose and muscle tissues [11,12].

**Figure 1:**
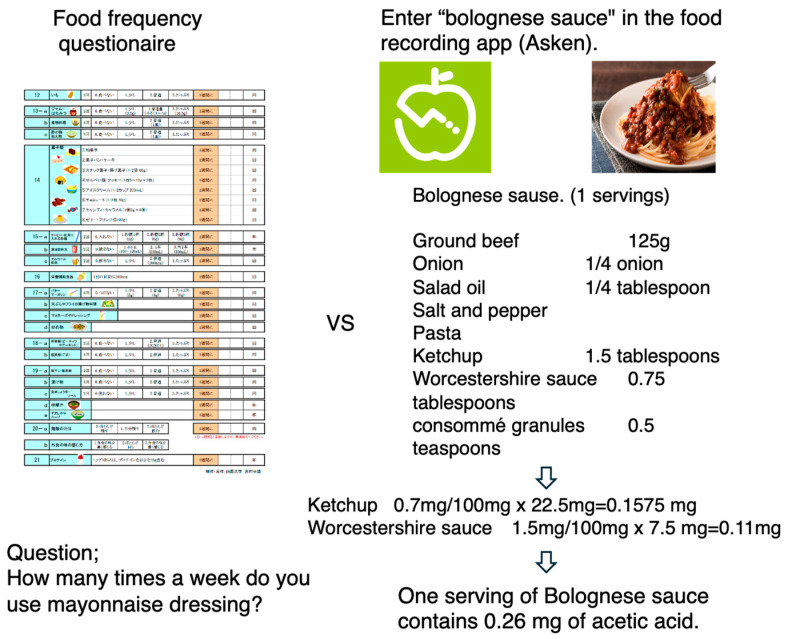
Comparison between food frequency questionnaire and food recoding app based on food recipe.

Diet assessment methods include weighed food records, estimated dietary records or food diaries, 24 h dietary recalls, food frequency questionnaires, and biomarker measurements [13,14]. For example, food frequency questionnaires generally assess only the frequency of vinegar, mayonnaise, and dressing intake [15,16]. Some questionnaires do not include questions about the use of seasonings [17]. However, acetic acid is used in many kinds of foods as a seasoning [18]. Moreover, determining the amount of acetic acid used based on appearance is difficult, and whether acetic acid is used as an ingredient in many dishes is not known. The use of acetic acid (vinegar) is therefore likely to be underestimated. Recently, food recording apps have become widely used in Japan. In these apps, the amount of nutrients is estimated by linking the name of a food to meal recipes in which it is included, providing nutrient and energy information [19,20] (Figure 1). Notably, meal recording apps are recipe based, which makes it easier to assess the amount of acetic acid used in foods.

A food frequency questionnaire estimates a question: “how many times a week do you use mayonnaise dressing?”. In contrast, a food recording app estimates the acetic acid content via analyzing recipe.

The aim of this study was to determine the relationships of acetic acid intake with protein and vitamin intake estimated via a food recording app (Asken). First, we checked the variation in the daily amount of acetic acid consumed for at least 7 days. We subsequently estimate the associations between the amount or frequency of acetic acid intake and metabolic parameters such as glycosylated hemoglobin (HbA1c) and lipid levels. Finally, we estimate the associations between the amount or frequency of acetic acid intake and the amount of energy intake and the intake of individual nutrients. 

## 2. Materials and Methods

### 2.1. Participants

**Figure 2:**
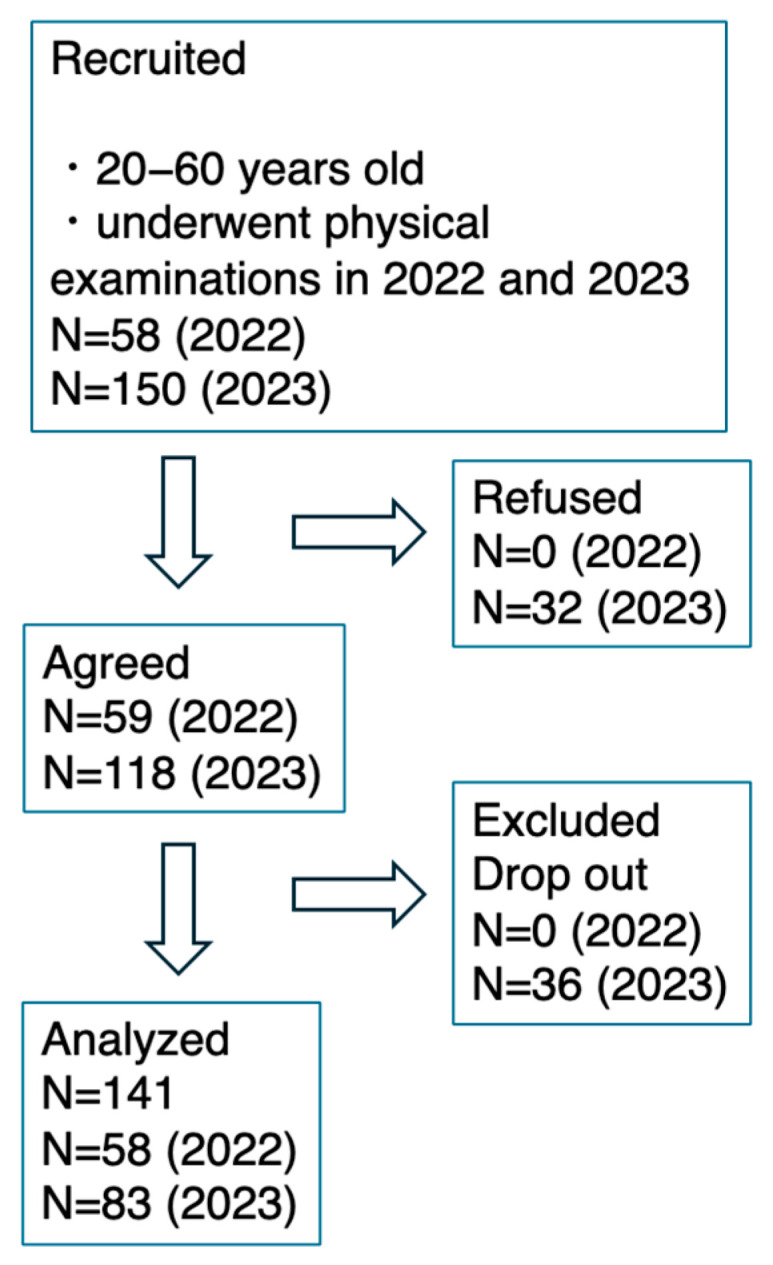
The inclusion and exclusion criteria for this study.

This was a retrospective cross-sectional study. Individuals who met the following criteria were recruited: underwent workplace health examinations at Fujita health university and were between the ages of 20 and 60 years. Fifty-eight and one hundred fifty individuals who underwent physical examinations in 2022 and 2023, respectively, were invited to participate in the study. At the study site, the participants were provided with an explanation of the nutrition app (Asken Inc., Tokyo, Japan) and were then asked to use the app to record the details of their meals between 1 and 31 December 2022 and between 1 and 31 December 2023. For one hundred forty-one individuals who kept records for >7 days, the 7-day mean values obtained via the app were obtained (Figure 2). The study was approved by the Ethics Committee of Fujita Health University (approval number: HM23-191, approved on 5 September 2023) (Figure 2).

### 2.2. Food Recording Application (Asken)

The Asken app allows for not only meal names but also photographs and barcodes to be manually input. Macronutrient and micronutrient contents can also be calculated as previously reported [19,20]. Our former study revealed a correlation coefficient of 0.9 for the relationship between Asken app data and data collected via a 1-day dietary record with respect to energy intake and the intake of the three macronutrients [19]. The nutritional value of food according to food recording apps is based on meal recipes. In the present study, we did not set any restrictions on these functions and allowed the participants to use their preferred method for recording dietary information. While meal recording applications are usually evaluated on a single-day basis, we used an average of at least seven days of records for our evaluation [20], and the data of participants who recorded their meals for >7 days were analyzed (Figure 1). For example, if you entered Bolognese sauce into the Asken app, the acetic acid content could be calculated from the amount of included ketchup and Worcestershire sauce, which are high in acetic acid, included in the recipe (Figure 1). In contrast, the questionnaire only asked about the frequency of mayonnaise and dressing consumption (Figure 1).

### 2.3. Statistical Analysis

The data were presented as the mean ± standard deviation (SD). The diurnal variation in the intake of each nutrient was represented as CV%. CV% was calculated by dividing the SD by the mean. Individuals were divided into four groups according to the quartile range of acetic acid intake: Q1 (0.027 ± 0.018 g), Q2 (0.080 ± 0.019 g), Q3 (0.16 ± 0.021 g), and Q4 (0.38 ± 0.27 g). The differences in each parameter among the four groups were estimated via the chi-square test or one-way ANOVA.

Next, we evaluated the associations between the amount or frequency of acetic acid intake and the amount of other nutrients consumed via multivariate analysis adjusted for age, BMI, sex, and energy intake. Data were presented as the mean (upper and lower limits of the 95% confidence interval). All the statistical analyses were conducted via SPSS software (version 28.0.0.0) (IBM Inc., Armonk, NY, USA). We considered *p* < 0.05 to indicate statistical significance.

## 3. Results

### 3.1. Large Variations in Acetic Acid Intake Were Observed for Most Nutrients

**Figure 3:**
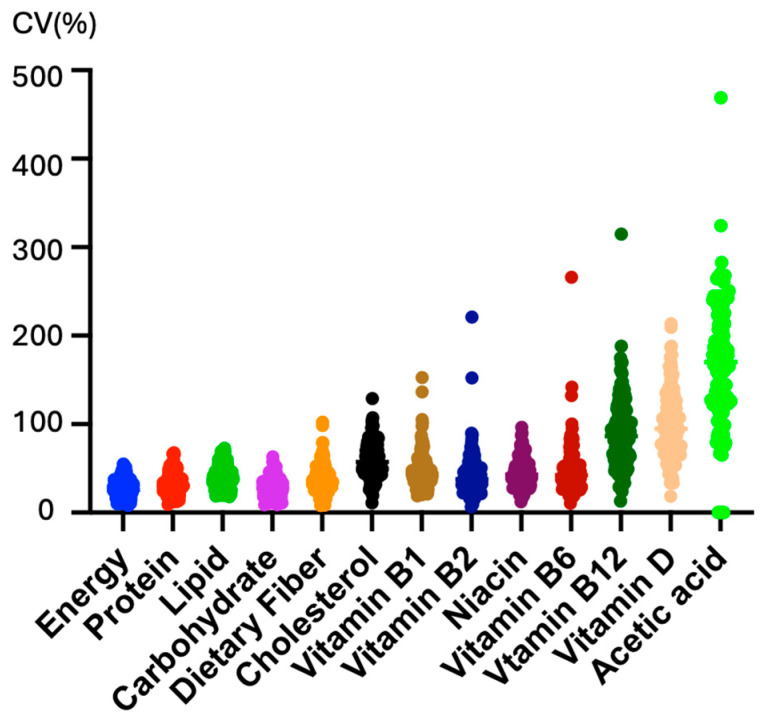
Diurnal variations in acetic acid intake. The coefficient of variation (%) for the intake of each nutrient, obtained from the dietary records, was calculated using the food recording app (Asken).

Using a food recording app (Asken), we estimated the amount of acetic acid intake for at least 7 days. The CV% of daily acetic acid intake was ten times greater than that of total energy intake and the intake of macronutrients such as carbohydrates, fat, and protein (Figure 3). 

The mean amount of acetic acid intake was 0.16 ± 0.19 g, and the mean frequency of acetic acid intake was 2.77 ± 1.66 days per week (Table 1). The participants were divided into four groups according to their daily acetic acid intake: Q1 (0.027 ± 0.018 g), Q2 (0.080 ± 0.019 g), Q3 (0.16 ± 0.021 g), and Q4 (0.38 ± 0.27 g) (*p* < 0.001) (Table 1). The frequencies of acetic acid intake in the Q1, Q2, Q3, and Q4 groups were 1.14 ± 0.81, 2.36 ± 0.98, 3.04 ± 1.28, and 4.67 ± 1.00 times per week, respectively (*p* < 0.001) (Table 1). There were 141 individuals who participated in this study (M:F ratio = 48:93). The mean age was 36.4 ± 11.1 years. The mean BMI was 21.7 ± 3.1 kg/m^2^, and the mean HbA1c level was 5.5 ± 0.6% (Table 1). The total cholesterol (T-Chol) and triglyceride (TG) levels were 189.0 ± 31.8 and 96.8 ± 74.0 mg/dL, respectively (Table 1). Except for the HDL-C level, there were no differences in age, sex, BMI, HbA1c level, T-Chol level, or TG level among the Q1–Q4 groups (Table 1). 

In terms of nutrient intake, energy, protein, lipid, carbohydrate, dietary fiber, saturated fatty acid, monounsaturated fatty acid (MUFA), polyunsaturated fatty acid (PUFA), and cholesterol intake were greater in the group with the highest acetic acid intake (Q4) (Table 2). For the Q1−Q4 groups, the mean intake values were as follows: the mean total energy intake was 1323.8 ± 327.0, 1409.7 ± 374.4, 1536.7 ± 406.5, and 1685.1 ± 371.2 kcal, respectively (*p* < 0.001); the mean protein intake was 50.8 ± 14.3, 54.4 ± 13.3, 59.7 ± 15.2, and 66.3 ± 17.3 g, respectively (*p* < 0.001); the mean fat intake was 48.5 ± 14.9, 53.5 ± 12.9, 57.5 ± 17.2, and 63.3 ± 14.6 g, respectively (*p* < 0.001); the mean carbohydrate intake was 177.1 ± 41.3, 183.1 ± 59.1, 198.7 ± 50.7, and 220.8 ± 54.0 g, respectively (*p* = 0.002); the mean dietary fiber intake was 13.5 ± 4.01, 14.4 ± 5.1, 15.1 ± 5.3, and 17.7 ± 4.2 g, respectively (*p* = 0.002); the mean saturated fatty acid intake was 14.2 ± 5.5, 16.0 ± 5.1, 16.1 ± 5.8, and 18.3 ± 5.1 g, respectively (*p* = 0.0018); the mean MUFA intake were 17.6 ± 5.9, 19.9 ± 4.8, 20.8 ± 7.2, and 23.3 ± 6.0 g, respectively (*p* = 0.001); the mean PUFA intake was 8.3 ± 2.7, 9.5 ± 2.5, 10.3 ± 3.1, and 12.3 ± 3.3 g, respectively (*p* < 0.001); and the mean cholesterol intake was 229.2 ± 94.8, 254.2 ± 94.1, 274.1 ± 111.3, and 309.2 ± 119.1 g, respectively (*p* = 0.014) (Table 2). Thus, as the amount of acetic acid intake increased, the amount of energy and macronutrient intake increased.

The differences in the levels of vitamin A, vitamin D, vitamin E, vitamin K, niacin, vitamin B6, vitamin B12, folate, pantothenic acid, and vitamin C among the Q1, Q2, Q3, and Q4 groups were significant (Table 3). For the Q1−Q4 groups, the mean intake values were as follows: the mean vitamin A intake was 361.3 ± 313.3, 382.6 ± 206.7, 542.0 ± 416.7, and 578.2 ± 396.3 g, respectively (*p* = 0.015); the mean vitamin E intake was 5.3 ± 1.9, 5.8 ± 2.0, 7.0 ± 4.0, and 9.8 ± 11.2 g, respectively (*p* = 0.011); the mean vitamin K intake was 134.5 ± 69.0, 160.4 ± 96.6, 179.6 ± 97.0, and 217.5 ± 114.2 g, respectively (*p* = 0.003); the mean niacin intake was 12.0 ± 4.3, 13.7 ± 3.4, 16.0 ± 6.3, and 17.2 ± 5.2 g, respectively (*p* < 0.001); the mean vitamin B12 intake was 3.5 ± 2.3, 4.3 ± 1.9, 5.0 ± 3.4, and 6.1 ± 3.5 g, respectively (*p* = 0.002); the mean folate intake was 196.4 ± 78.6, 216.6 ± 98.4, 236.5 ± 94.2, and 280.7 ± 91.1 g, respectively (*p* = 0.001); the mean pantothenic acid intake was 3.9 ± 1.4, 4.2 ± 1.5, 4.9 ± 2.1, and 5.5 ± 1.9 g, respectively (*p* < 0.001); and the mean vitamin C intake was 69.2 ± 38.7, 67.3 ± 43.1, 71.8 ± 41.7, and 102.1 ± 51.0 g, respectively (*p* = 0.003) (Table 3). Thus, as acetic acid intake increases, the intake of many vitamins increases.

### 3.2. Associations between Acetic Acid Intake and Metabolic Parameters

Next, we examined the associations between the amount or frequency of acetic acid intake and metabolic parameters. After adjusting for age, sex, BMI, and energy, metabolic parameters (HbA1c, cholesterol-Chol, TG, and HDL-C levels) were not significantly associated with the amount of acetic acid intake (HbA1c: β = −0.12 (−0.58, 0.35), *p* = 0.62; T-Chol: β = 18.71 (−6.94, 44.36), *p* = 0.15; TGs: β = 20.09 (−39.08, 79.25), *p* = 0.5; HDL-C: β= −3.88 (−15.06, 7.30), *p* = 0.49) (Table 4). The metabolic parameters (HbA1c, T-Chol, TG, and HDL-C levels) were also not significantly associated with the frequency of acetic acid intake (HbA1c: β = 0.029 (−0.026, 0.085), *p* = 0.3; T-Chol: β = −1.66 (−4.77, 1.45), *p* = 0.29; TGs: β = 3.00 (−4.14, 10.14), *p* = 0.41; HDL-C: β = −0.86 (−2.21, 0.48), *p* = 0.21) (Table 4). Thus, neither the amount nor the frequency of acetic acid intake was associated with metabolic parameters.

Next, we performed a multivariate analysis of the relationships between the amount of acetic acid intake and the amount of daily energy and nutrient intake. Acetic acid intake was associated with protein, carbohydrate, dietary fiber, PUFA, and cholesterol intake (Table 5). After adjusting for age, BMI, sex, and energy intake, the amount of acetic acid intake was significantly associated with the amount of daily protein and cholesterol intake (protein: β = 11.9 (5.1, 18.6), *p* < 0.001; cholesterol: β = 80.7 (4.5, 156.9), *p* = 0.04) (Table 5). In contrast, the frequency of acetic acid intake was associated with the amount of protein, MUFA, and PUFA intake (protein: β = 3.83 (2.33, 5.33), *p* < 0.001; MUFAs: β = 1.43 (0.84, 2.03), *p* < 0.001; PUFAs: β = 0.94 (0.65, 1.23), *p* < 0.001) (Table 4). Similarly, after adjusting for age, BMI, sex, and energy intake, the frequency of acetic acid intake was associated with the amount of protein, MUFA, and PUFA intake (protein: β = 1.04 (0.20, 1.87), *p* = 0.015; MUFAs: β = 0.41 (0.037, 0.79), *p* = 0.031; PUFAs: β = 0.44 (0.25, 0.63), *p* < 0.001) (Table 5).

The amount of acetic acid intake was significantly associated with the levels of vitamin A, vitamin D, vitamin E, vitamin B1, vitamin B2, niacin, vitamin B6, vitamin B12, folate, pantothenic acid, and vitamin C (Table 6). After adjusting for age, sex, BMI, and energy intake, the amount of acetic acid intake was also significantly associated with the frequency of vitamin A, vitamin D, vitamin E, vitamin B1, vitamin B2, niacin, vitamin B6, vitamin B12, folate, pantothenic acid, and vitamin C intake (A: β = 371.5 (84.1, 658.9), *p* = 0.012, D: 8.3 (5.1, 11.5), *p* < 0.001, E: 5.2 (0.02, 10.3), *p* = 0.049, B1: 7.1 (5.5, 8.6), *p* < 0.001, B2: 4.3 (3.3, 5.2), *p* < 0.001, niacin; 9.0 (5.5, 12.4), *p* < 0.001, B6: 3.9 (2.7, 5.0), *p* < 0.001, B12: 4.0 (1.5, 6.5, *p* < 0.001)) (Table 6). Similarly, the frequency of acetic acid intake was significantly associated with the frequency of vitamin A, vitamin D, vitamin E, vitamin K, vitamin B1, vitamin B2, niacin, vitamin B6, vitamin B12, folate, pantothenic acid, and vitamin C intake. After adjusting for age, sex, BMI, and energy intake, the amount of acetic acid intake was also significantly associated with the frequency of vitamin B1, vitamin B12, and niacin intake (B1: β = 0.3 (0.02, 0.5), *p* = 0.031; niacin: β = 0.5 (0.04, 1.0), *p* = 0.032; and B12: β = 0.4 (0.1, 0.7), *p* = 0.008) (Table 6). Thus, both the amount and frequency of acetic acid intake were significantly associated with the amount of vitamin B1, niacin, and vitamin B12 intake.

## 4. Discussion

Previous food frequency questionnaires have predicted acetic acid intake based on the amount of dressing or mayonnaise you put on your food and have therefore failed to reveal the amount of acetic acid intake. The present study assessed the amount of acetic acid in foods other than seasonings (mayonnaise, dressings), which is not captured by traditional questionnaires, via a food recording application. Acetic acid intake was highly variable across days, similar to the intake of vitamin D and vitamin B12. This finding implied the importance of investigating acetic acid intake over a seven-day period, which was not possible with conventional questionnaires. We subsequently assessed the associations between acetic acid intake and metabolic parameters. Increased acetic acid intake was related to an increased intake of energy, macronutrients, and many vitamins but was not related to BMI or HbA1c, T-Chol, or TG levels. After adjusting for energy intake, sex, age, and BMI, the amount of acetic acid intake was compared with metabolic parameters, but no associations were found. On the other hand, after adjusting for energy intake, sex, age, and BMI, an association was found between acetic acid intake and the intake of protein, cholesterol, and many vitamins except vitamin K. In relation to the frequency of acetic acid intake, acetic acid intake was positively associated with protein, MUFA, PUFA, vitamin B1, niacin, and vitamin B12 intake. Thus, the amount and frequency of acetic acid intake were shown to be associated with protein intake, vitamin B1 and B12 intake, and niacin intake regardless of the amount of energy intake, sex, age, or BMI. The results of this study are the first to identify that acetic acid intake is associated with the intake of protein and vitamins such as vitamin B1, niacin, and vitamin B12.

In this study, we first determined that acetic acid intake was highly variable across days. Acetic acid and sodium acetate are included in many food products, but the amount of sodium acetate used as a food additive is considered the minimum necessary amount [21]. In contrast, the amount of acetic acid in vinegar is approximately 4–5 g per 100 g, so a spoonful (15 mL) of vinegar contains approximately 0.75 g of acetic acid [18]. Ketchup and mayonnaise contain 0.7 g and 0.5 g acetic acid per 100 g, respectively [18]. Since large amounts of acetic acid are included in seasonings, such as sauces and dressings, dressings and mayonnaise are included in food frequency questionnaires. However, the seasonings used in cooked foods are not known. Moreover, we cannot ignore the amount of acetic acid present in many foods. Therefore, the evaluation of acetic acid intake via food recording applications that estimate acetic acid intake from meal recipes used in this study is a more useful method. More recipes for foods included in meal recording apps would lead to a more accurate assessment of acetic acid intake.

We also revealed that acetate intake was not significantly associated with metabolic parameters. No associations between metabolic parameters (HbA1c and blood lipid levels) and the amount of acetic acid intake have been reported. One of the reasons for this is that the questionnaire included only the amount of dressing or mayonnaise they used, so it was not possible to measure their acetic acid intake accurately. Moreover, studies of improved glucose tolerance have used larger daily doses of acetic acid (0.75 g), and it is likely that the amount of acetic acid normally ingested is too small to affect the effects of acetic acid on glucose and lipid metabolism. In fact, the mean amount of acetic acid consumed in the Q4 group was 0.37 g, which was much lower than the amount (0.75 g) of acetic acid that has been reported to have anti-obesity effects. Considering that a person with a normal diet consumes only approximately half a tablespoon of acetic acid at most, studies including a larger number of people are needed to verify the beneficial effects of acetic acid consumption on health. Notably, our data do not include people whose acetic acid intake was high enough to affect glucose tolerance in this study.

In this study, as the amount of acetic acid intake increased, the amount of energy intake and vitamin intake increased. The amount of acetic acid intake was associated with the amount of protein and all vitamins except vitamin K consumed, independent of age, sex, BMI, and total energy intake. These findings are consistent with the use of acetic acid for seasoning. Vinegar and other sour seasonings are known to stimulate saliva secretion and increase appetite. Sour seasonings such as vinegar, ketchup, and mayonnaise are widely used in the preparation of meat and fish. Therefore, it is reasonable that increased acetic acid intake was associated with an increased intake of protein and vitamins.

We also estimated the association between the frequency of acetic acid intake and the amount of the intake of individual nutrients. The results were partially consistent with those found for acetic acid intake. In particular, protein, vitamin B1, niacin, and vitamin B12 intake was associated not only with the amount of acetic acid intake but also with the frequency of acetic acid intake. The results were partially consistent with those found for acetic acid intake. In particular, protein, vitamin B1, niacin, and vitamin B12 intake was related not only to the amount of acetic acid intake but also to the frequency of acetic acid intake. In other words, even if acetic acid intake is not known, protein intake and the intake of some vitamins can be analogized if the frequency of acetic acid intake is known. A biomarker that could assess acetic acid intake would be useful for determining protein and vitamin intake. However, it is difficult to estimate acetic acid intake via biomarkers such as blood acetic acid. Acetate is also produced by the microbiota in the colon, and glucose and ethanol are converted to acetate in the liver. One method to estimate acetic acid intake might be to assign scores to foods high in acetic acid so that people can easily evaluate their intake of not only acetic acid but also protein and vitamins.

A limitation of this study is that it was a cross-sectional observational study, and causal relationships are not known. In addition, because this was an exploratory study, the number of participants was small, and the sex ratio was skewed. The participants were primarily women, with a 1:2 male-to-female ratio. One possible reason is that our workplaces overwhelmingly have twice as many women as men, although previous studies have also included more female participants [19,20]. As described in the discussion, more participants are needed to test the effects of acetic acid on the development of lifestyle-related diseases, such as obesity and diabetes mellitus. In addition, most of the participants in this study were health checkup recipients and had not been diagnosed with abnormalities such as lipid abnormalities, diabetes, or obesity. Therefore, future studies are needed to determine whether the same results can be obtained in people with underlying diseases.

## 5. Conclusions

In conclusion, the use of a recipe-based meal recording application allowed for a more extensive evaluation of the acetic acid content of foods. Our study revealed significant diurnal variations in acetic acid intake, and increased acetic acid intake was positively associated with increased protein and vitamin intake, independent of dietary energy intake, age, sex, and BMI. The use of seasonings containing acetic acid, such as vinegar, may be beneficial for the intake of a variety of vitamins as well as proteins. In the future, questions about the amount and frequency of the intake of acetic acid-rich foods may provide an indication of whether protein and vitamin intake is adequate.

## Figures and Tables

**Table 1:** Background characteristics of the individuals.

	Total	Q1	Q2	Q3	Q4	
	n = 141	n = 36	n = 35	n = 35	n = 35	
Acetic acid intake per a day	0.16 (0.19)	0.027 (0.018)	0.080 (0.019)	0.16 (0.021)	0.38 (0.27)	***p* < 0.001**
Days per week to eat foods containing acetic acid	2.77 (1.66)	1.14 (0.81)	2.36 (0.98)	3.04 (1.28)	4.67 (1.00)	***p* < 0.001**
Sex (male)	48 (34%)	15 (42%)	11 (31%)	12 (34%)	10 (29%)	NS
Age (yo)	36.4 (11.1)	34.5 (11.8)	37.8 (11.1)	34.7 (10.2)	38.7 (11.0)	NS
Height (cm)	163.1 (6.6)	163.1 (7.9)	162.4 (7.8)	162.5 (7.9)	164.2 (6.6)	NS
BW (kg)	58.1 (11.0)	58.4 (10.7)	55.6 (8.9)	58.7 (14.5)	59.6 (9.1)	NS
BMI	21.7 (3.1)	21.9 (3.0)	21.0 (1.9)	22.1 (4.1)	22.1 (2.9)	NS
HbA1c (%)	5.5 (0.6)	5.5 (0.5)	5.4 (0.3)	5.6 (0.9)	5.5 (0.3)	NS
T-Chol (mg/dL)	189.04 (31.82)	182.4 (32.7)	195.7 (26.4)	187.9 (33.5)	190.4 (34.0)	NS
TG (mg/dL)	96.84 (73.99)	94.4 (54.9)	82.4 (51.4)	106.9 (100.5)	103.8 (79.4)	NS
HDL-C (mg/dL)	63.02 (14.41)	58.6 (13.6)	68.6 (15.9)	62.2 (15.1)	62.8 (11.4)	***p* = 0.03**

NS means not significant. Bold letter means significant. Bold letter means significant.

**Table 2:** The summary of nutrient intake in the participants.

	Total	Q1	Q2	Q3	Q4	
	n = 141	n = 36	n = 35	n = 35	n = 35	
Acetic acid	0.16 (0.19)	0.027 (0.018)	0.080 (0.019)	0.16 (0.021)	0.38 (0.27)	***p* < 0.001**
Total energy	1487.4 (391.4)	1323.8 (327.0)	1409.7 (374.4)	1536.7 (406.5)	1685.1 (371.2)	***p* < 0.001**
Protein	57.75 (16.03)	50.82 (14.34)	54.35 (13.28)	59.69 (15.15)	66.33 (17.28)	***p* < 0.001**
Fat	55.66 (15.79)	48.50 (14.87)	53.50 (12.89)	57.49 (17.22)	63.34 (14.59)	***p* < 0.001**
Carbohydrate	194.8 (53.8)	177.1 (41.3)	183.1 (59.1)	198.7 (50.7)	220.8 (54.0)	***p* = 0.002**
Dietary fiber	15.15 (4.90)	13.50 (4.01)	14.39 (5.11)	15.11 (5.31)	17.65 (4.22)	***p* = 0.002**
Saturated FA	16.11 (5.51)	14.17 (5.45)	15.97 (5.07)	16.08 (5.80)	18.26 (5.10)	***p* = 0.018**
MUFA	20.40 (6.32)	17.62 (5.88)	19.93 (4.84)	20.80 (7.21)	23.34 (6.01)	***p* = 0.001**
PUFA	10.09 (3.24)	8.33 (2.69)	9.49 (2.50)	10.28 (3.14)	12.31 (3.31)	***p* < 0.001**
Cholesterol	266.4 (108.2)	229.2 (94.8)	254.2 (94.1)	274.1 (111.3)	309.2 (119.1)	***p* = 0.014**

Bold letter means significant.

**Table 3:** The summary of vitamin intake in the participants.

	Total	Q1	Q2	Q3	Q4	
	n = 141	n = 36	n = 35	n = 35	n = 35	
Vitamin A	465.30 (352.95)	361.33 (313.31)	382.64 (206.70)	541.97 (416.67)	578.23 (396.30)	***p* = 0.015**
Vitamin D	5.3 (3.9)	4.3 (3.0)	4.8 (3.2)	5.6 (4.6)	6.6 (4.2)	NS
Vitamin E	6.98 (6.26)	5.34 (1.92)	5.83 (1.98)	6.99 (3.95)	9.81 (11.20)	***p* = 0.011**
Vitamin K	172.71 (98.94)	134.47 (69.01)	160.35 (96.58)	179.63 (97.01)	217.48 (114.21)	***p* = 0.003**
Vitamin B1	1.10 (2.10)	0.79 (0.30)	0.82 (0.24)	0.96 (0.44)	1.85 (4.12)	NS
Vitamin B2	1.12 (1.32)	0.89 (0.55)	0.91 (0.40)	1.07 (0.59)	1.62 (2.45)	NS
Niacin	14.68 (5.27)	11.97 (4.33)	13.69 (3.40)	15.96 (6.31)	17.17 (5.18)	***p* < 0.001**
Vitamin B6	1.29 (1.46)	1.18 (1.41)	0.96 (0.35)	1.25 (0.91)	1.78 (2.32)	NS
Vitamin B12	4.73 (3.0)	3.51 (2.25)	4.30 (1.93)	5.02 (3.39)	6.13 (3.51)	***p* = 0.002**
Folate	232.3 (95.1)	196.4 (78.6)	216.6 (98.4)	236.5 (94.2)	280.7 (91.1)	***p* = 0.001**
Pantothenic acid	4.61 (1.83)	3.90 (1.43)	4.20 (1.48)	4.87 (2.05)	5.49 (1.91)	***p* < 0.001**
Vitamin C	77.52 (45.63)	69.22 (38.67)	67.26 (43.12)	71.79 (41.72)	102.06 (50.97)	***p* = 0.003**

NS means not significant. Bold letter means significant.

**Table 4:** Multivariate analysis of acetic acid intake and metabolic parameters adjusted with age, sex, BMI, and energy intake.

	Amount of Acetic Acid Intake				Frequency of Acetic Acid Intake			
	Unadjusted		Adjusted		Unadjusted		Adjusted	
Outcome	β	*p*	Β	*p*	β	*p*	β	*p*
HbA1c	0.19 (−0.31, 0.68)	**<0.001**	−0.12 (−0.58, 0.35)	0.62	0.052 (−0.004, 0.11)	0.07	0.029 (−0.026, 0.085)	0.3
T-Chol	31.90 (4.26, 59.53)	**0.024**	18.71 (−6.94, 44.36)	0.15	0.082 (−3.15, 3.32)	0.96	−1.66 (−4.77, 1.45)	0.29
TG	55.12 (−9.68, 119.92)	0.095	20.09 (−39.08, 79.25)	0.5	5.31 (−2.2, 12.78)	0.16	3.00 (−4.14, 10.14)	0.41
HDL-C	−5.00 (−17.72, 7.72)	0.44	−3.88 (−15.06, 7.30)	0.49	−0.53 (−1.99, 0.93)	0.48	−0.86 (−2.21, 0.48)	0.21

Bold letter means significant.

**Table 5:** Multivariate analysis of acetic acid intake and nutrient intake adjusted with age, sex, BMI, and energy intake.

	Amount of Acetate Intake				Frequency of Acetate Intake			
	Unadjusted		Adjusted		Unadjusted		Adjusted	
Outcome	β	*p*	Β	*p*	β	*p*	β	*p*
Energy	434.5 (96.0, 773.1)	**0.012**	ND	ND	83.62 (46.39, 120.84)	**<0.001**	ND	ND
Protein	25.7 (12.2, 39.2)	**<0.001**	11.9 (5.1, 18.6)	**<0.001**	3.83 (2.33 ,5.33)	**<0.001**	1.04 (0.20, 1.87)	**0.015**
Lipid	13.6 (−0.2, 27.3)	0.054	−2.1 (−8.6, 4.5)	0.53	3.89 (2.42, 5.35)	**<0.001**	0.97 (0.20, 1.75)	**0.015**
Carbohydrate	56.3 (9.6, 103.0)	**0.018**	0.6 (−17.5, 18.7)	0.95	9.42 (4.19, 14.66)	**<0.001**	−1.40 (−3.57, 0.78)	0.21
Dietary Fiber	4.7 (0.5, 9.0)	**0.03**	1.0 (−2.1,4.1)	0.53	0.83 (0.36, 1.31)	**<0.001**	0.11 (−0.27, 0.49)	0.56
SFA	3.3 (−1.6, 8.1)	0.18	−1.2 (−4.4, 2.0)	0.45	0.93 (0.39, 1.46)	**<0.001**	−0.01 (−0.39, 0.38)	0.96
MUFA	5.0 (−0.5, 10.5)	0.08	−0.2 (−3.4, 2.9)	0.88	1.43 (0.84, 2.03)	**<0.001**	0.41 (0.037, 0.79)	**0.031**
PUFA	4.3 (1.5, 7.0)	**0.003**	1.6 (−0.1, 3.2)	0.064	0.94 (0.65, 1.23)	**<0.001**	0.44 (0.25, 0.63)	**<0.001**
Cholesterol	153.9 (61.7, 246.1)	**0.001**	80.7 (4.5, 156.9)	**0.04**	22.73 (12.41, 33.04)	**<0.001**	7.97 (−1.28, 17.23)	0.09

Bold letter means significant. ND means not determined.

**Table 6:** Multivariate analysis of acetic acid intake and vitamin intake adjusted with age, sex, BMI, and energy intake.

	Amount of Acetate Intake (g)				Frequency of Acetate Intake (Days)			
	Unadjusted		Adjusted		Unadjusted		Adjusted	
Outcome	Β	*p*	β	*p*	β	*p*	β	*p*
Vitamin A	507.1 (206.7, 807.6)	**0.001**	371.5 (84.1, 658.9)	**0.012**	37.8 (2.5, 73.1)	**0.036**	4.15 (−31.4, 39.7)	0.817
Vitamin D	8.8 (5.7, 11.9)	**<0.001**	8.3 (5.1, 11.5)	**<0.001**	0.5 (0.09, 0.9)	**0.016**	0.29 (−0.13, 0.71)	0.171
Vitamin E	8.0 (2.6, 11.9)	**0.004**	5.2 (0.02, 10.3)	**0.049**	0.83 (0.2, 1.5)	**0.009**	0.27 (−0.36, 0.90)	0.393
Vitamin K	156.0 (19.1, 190.6)	**0.017**	57.0 (−27.4, 134.5)	0.15	17.5 (7.9, 27.1)	**<0.001**	8.7 (−0.7, 18.0)	0.68
Vitamin Β1	6.7 (5.2, 13.4)	**<0.001**	7.1 (5.5, 8.6)	**<0.001**	0.2 (0.01, 0.4)	**0.037**	0.3 (0.02, 0.5)	**0.031**
Vitamin B2	4.2 (3.3, 5.2)	**<0.001**	4.3 (3.3, 5.2)	**<0.001**	0.2 (0.03,0.3)	**0.018**	0.044 (−0.035, 0.12)	0.276
Niacin	11.7 (7.4, 11.9)	**<0.001**	9.0 (5.5, 12.4)	**<0.001**	1.1 (0.6, 1.6)	**<0.001**	0.5 (0.04, 1.0)	**0.032**
Vitamin B6	3.9 (2.8, 5.0)	**<0.001**	3.9 (2.7, 5.0)	**<0.001**	0.2 (0.011, 0.30)	**0.035**	0.12 (−0.04, 0.3)	0.134
Vitamin B12	5.1 (2.6, 7.6)	**<0.001**	4.0 (1.5, 6.5)	**0.002**	0.6 (0.3, 0.9)	**<0.001**	0.4 (0.1, 0.7)	**0.008**
Folate	173.2 (94.2, 252.2)	**<0.001**	119.1 (50.6, 187.7)	**<0.001**	15.2 (5.9, 24.6)	**0.002**	3.1 (−5.5, 11.8)	0.473
Pantothenic acid	3.4 (1.9, 4.9)	**<0.001**	2.3 (1.1, 3.5)	**<0.001**	0.3 (0.1, 0.5)	**<0.001**	0.07 (−0.1, 0.2)	0.36
Vitamin C	78.0 (39.8, 116.2)	**<0.001**	54.7 (18.3, 91.1)	**0.004**	5.7 (1.2, 10.2)	**0.014**	0.7 (−3.9, 5.2)	0.763

Bold letter means significant.

## Data Availability

Some or all datasets generated during and/or analyzed during the current study are not publicly available but are available from the corresponding author upon reasonable request.
